# Microbiota management: a perspective for kidney transplant patients

**DOI:** 10.3389/frtra.2026.1703048

**Published:** 2026-02-19

**Authors:** Cláudia Silva Souza, Niels Olsen Saraiva Camara, Thaís Alves-Silva

**Affiliations:** 1 Transplantation Immunobiology Lab, Department of Immunology, Institute of Biomedical Sciences, University of São Paulo, São Paulo, Brazil; 2 Clinical and Experimental Immunology and Nephrology Lab, Division of Nephrology, Department of Medicine, Federal University of São Paulo, São Paulo, Brazil

**Keywords:** chronic kidney disease (CKD), gut-kidney aixs, high-fiber diets, kidney transplantation, prebiotics, probiotic, synbiotic

## Abstract

Chronic kidney disease (CKD) is recognized as one of the most significant public health issues globally, with approximately 40% of patients progressing to end-stage kidney disease (ESKD). Transplantation remains the most indicated option for many patients with ESKD; however, these individuals frequently experience hospital readmissions and face a heightened risk of developing other complications. Although short-term allograft survival, typically around one year, tends to be satisfactory, long-term graft survival is often compromised due to acute and chronic rejection, which can be mediated by antibodies or other recipient-related factors. Recent studies and review articles have emphasized the relationship between intestinal dysbiosis and chronic renal failure, as well as the poor outcomes associated with kidney transplantation. The CKD itself, as well as the immunosuppressive medications used by organ transplant recipients, can lead to intestinal dysbiosis, which in turn increases the production of uremic toxins that can harm even the transplanted kidney. Clinical studies have demonstrated that the combination of prebiotics, probiotics, and synbiotics—supplements that aid in the establishment of intestinal microbiota—can effectively help control the production of nitrogenous substances, reduce renal inflammation, and alleviate gastrointestinal symptoms in patients with CKD. Experimental models have shown that short-chain fatty acids derived from gut fermentation of dietary fiber, such as butyrate and acetate, could improve renal function and reduce renal inflammation, allowing better acceptance of kidney allograft, partly by inducing T regulatory cells. Despite the growing evidence supporting the positive effects of maintaining a balanced microbiota, there is still a lack of comprehensive reviews in this field. Additionally, recent findings suggest that the type of fiber consumed may influence intestinal health and even increase susceptibility to colorectal cancer, depending on the fiber type. Therefore, we aim to explore how substances derived from gut fermentation of dietary fiber, in addition to probiotics, prebiotics, and synbiotics, contribute to allograft tolerance, with a particular focus on their potential application in establishing renal function in allograft recipients.

## Introduction

CKD is a condition characterized by a progressive decline in kidney function. This condition is defined by the presence of kidney damage, which can be identified through histopathological abnormalities, abnormal urinary sediment, or increased rates of urinary albumin excretion. Additionally, CKD could also be classified by an estimated glomerular filtration rate (eGFR) of less than 60 mL/min/1.73 m², maintained for three months or longer (1–3).

CKD presents a significant global public health challenge, as it affects more than 10% of the worldwide population (4–6), and it has the potential to advance to kidney failure, referred to as ESKD. Approximately 40% of CKD patients progress to this stage (5, 6), which frequently results in renal replacement therapy, which includes renal dialysis and kidney transplantation (7–9).

Recent studies have highlighted the gut-kidne*y* axis, showcasing a bidirectional relationship between kidney function and intestinal microbiota (10). However, the mechanisms involved in this relationship remain poorly understood. The intestinal microbiota comprises a complex and extensive community of microorganisms, including bacteria, archaea, protists, fungi, and viruses (11). This diverse ecosystem of gut microbes plays essential roles in numerous physiological processes, supporting the maintenance of cellular functions and overall health (11). Under homeostatic conditions, these microorganisms coexist symbiotically in the gut, producing vital metabolites, such as vitamins and short-chain fatty acids (SCFAs), which help maintain intestinal barrier integrity (12).

When there is an imbalance in microbiota composition—known as dysbiosis—there tends to be a decrease in SCFA-producing bacteria and an increase in pathogenic bacteria, resulting in elevated toxin production (13, 14). SCFAs have immunomodulatory effects, promoting intestinal and systemic immune tolerance by developing regulatory T cells (Treg) and producing anti-inflammatory cytokines (15, 16). These actions significantly contribute to maintaining intestinal barrier integrity. Conversely, during dysbiosis, there is an increase in endotoxin production, such as lipopolysaccharides (LPS), which induce an inflammatory environment by activating inflammatory pathways like NF-kB and generating inflammatory cytokines, such as monocyte chemoattractant protein-1 (MCP-1), interleukin-6 (IL-6), and tumor necrosis factor (TNF-α) (17–19). This process recruits inflammatory cells and heightens intestinal permeability, contributing to a condition often referred to as “leaky gut” (17, 19), that is associated with the reduced expression of tight-junction proteins (19). The released endotoxins frequently enter the bloodstream, resulting in systemic inflammation and disrupting overall metabolism.

Dysbiosis is often associated with cardiometabolic diseases and diets low in fiber and high in animal protein (20, 21). These diets are correlated with a reduction in SCFA-producing bacteria and an increase in sulfate products, such as indoxyl-sulfate and cresyl-sulfate (20), which enter the bloodstream due to “leaky gut” and may damage tubular epithelial cells (22). Although the mechanisms underlying the gut-kidne*y* axis are not completely understood, dysbiosis in patients with CKD is typically linked to a decrease in beneficial bacteria, such as *Lactobacillus*, *Prevotella,* and *Bifidobacteria*, along with an increase in pathogenic or opportunistic bacteria such as *Pseudomonadota* (previously *Proteobacteria*), *Enterococcus, Hungatella, Enterocloster,* and *Desulfovibrio* species (23–25).

Additionally, metabolic acidosis, a decreased glomerular filtration rate, and a low-fiber diet—often observed in CKD patients—are associated with reduced clearance of sulfate products and urea nitrogen toxins (26–30), which are normally excreted in urine. However, in CKD, there is an accumulation of uremic toxins in the bloodstream due to increased synthesis by gut microbiota and reduced clearance. This accumulation harms tubular epithelial cells and disrupts the integrity of the intestinal epithelial barrier, perpetuating the imbalance in intestinal microbiota. As a result, there is a vicious cycle between the injured kidney and the “leaky gut”, contributing to the progression of kidney disease, which often culminates in the need for kidney transplantation.

To address gut microbiota imbalances and potentially restore balance in kidney transplant patients, the use of prebiotics, probiotics, and synbiotics has increased. Prebiotics are types of fiber that the human body cannot digest, but they can be broken down and utilized by the microorganisms in our gut. Probiotics, on the other hand, are live microorganisms that help in digesting food. Synbiotics are a combination of both prebiotics and probiotics.

Research on these interventions in patients with chronic renal failure and those who have undergone solid organ transplants has grown significantly. However, despite the growing evidence supporting the benefits of a balanced microbiota, there is a lack of comprehensive reviews specifically addressing prebiotics, probiotics, and synbiotics in kidney transplant patients.

Therefore, our aim is to provide a thorough and up-to-date overview of the role of intestinal microbiota and how it can be modulated through prebiotics, probiotics, and synbiotics as preventive, therapeutic, or supplementary strategies for various types of transplants, particularly kidney transplants.

## Gut microbiota: overview of intestinal, immune cells, and renal system effects

The gut microbiota (GM) encompasses the highest density of microorganisms in the organism and is therefore also the most investigated and characterized (31–34). It is composed of countless microorganisms, including fungi, archaea, bacteria, and viruses; however, predominantly formed by bacteria (31, 35). In early life the GM is mainly home to aerobic or facultative anaerobic bacteria of the genera *Enterobacter*, *Enterococcus*, and *Staphylococcus*. During development and adulthood life, with changes in the intestinal microenvironment, influenced mainly by environmental factors (36, 37), anaerobic bacteria proliferate, including those of the genera *Bifidobacterium, Clostridium, and Bacteroides* (32–34, 38). Simultaneously, the influence of an anaerobic microbiota is quite favorable, given its important role in aiding digestion as well as synthesizing substances beneficial to the body (39, 40). Furthermore, anaerobic microbiota contribute to host defense by preventing colonization by pathogens (40). Anaerobic microorganisms perform these functions by binding to cell adhesion receptors, competing for nutrients, and generating antimicrobial substances, such as the end products of fermentation (40).

Among the substances derived from bacterial fermentation, SCFAs, produced mainly in the colon from the fermentation of dietary fiber, have attracted considerable attention due to their high immunogenic potential (41, 42). In this sense, studies have extensively demonstrated that SCFAs, including acetate, butyrate, and propionate, in addition to being important nutritional sources for colonic epithelial cells, act directly in maintaining an anaerobic intestinal environment by controlling the proliferation of facultative anaerobic pathogens and the epithelial layer of the intestinal barrier (21, 43, 44). Furthermore, these microbial metabolites perform the important function of regulating intestinal and systemic immunity, contributing to the body's immune homeostasis (45).

Once they enter the bloodstream, SFCAs act on various cells in the human body by activating their G protein-coupled transmembrane receptors (GPCRs - GPR41, GPR43, GPR109A, and olfactory receptor 78) and inhibiting histone deacetylase (HDAC) enzymes (46). In this sense, it has been demonstrated that SCFAs regulate antigen presentation by dendritic cells, consequently interfering with the differentiation and function of CD4^+^ and CD8^+^ T cells (38, 47, 48), control the extrathymic differentiation of Treg (49), they induce the migration of neutrophils to the inflammatory environment and increase their phagocytic capacity (50). They inhibit the production of pro-inflammatory cytokines by macrophages and contribute to the reprogramming of macrophage polarization to an M2 (anti-inflammatory) phenotype (51) and induce B cell differentiation and IgA secretion by these cells (44).

In addition to their intestinal and immune cells effects, SCFAs have also been reported as determinants in modulating kidney health and disease (52). The numerous benefits of SFACs produced by gut bacteria for kidney health include reducing inflammation, oxidative stress, renal fibrosis, and slowing the progression of CKD (53–56). Despite this, external factors, including changes in diet, use of antibiotics and other medications, chronic stress, and pathogenic infections, can lead to an imbalance in the gut microbiota, giving rise to a condition called intestinal dysbiosis (52, 57). This clinical presentation is characterized by a reduction in the quantity and diversity of beneficial bacteria and an exorbitant increase in harmful bacteria in the gastrointestinal tract (37). Several studies have shown that exacerbated dysbiosis is associated with the development of acute kidney injury (AKI) (58, 59) and CKD (59).

Additionally, it has been widely demonstrated that this clinical situation is associated with the development of numerous other comorbidities such as obesity (47, 60, 61), cardiovascular disease (CVD) (62), inflammatory bowel disease (63), hepatic steatosis (64), insulin resistance and type 2 diabetes mellitus (57, 65) and cognitive impairment (57). Considering the points raised by these studies, intestinal dysbiosis can lead to the occurrence of these events, among other factors, by repressing metabolic pathways and consequently reducing the secretion of beneficial metabolites produced by these microorganisms, such as SCFAs, while increasing the concentration of endotoxins in the bloodstream. Thus, taken together, these results reinforce the perspective that abrupt changes in the gut microbiota in adulthood are closely associated with programmatic immune and renal outcomes, contributing to the individual's health and disease pattern.

## Microbiota modulation as an additive to conventional therapies

GM houses a wide variety of microorganisms that, in a state of equilibrium, act in a coordinated manner by exercising numerous beneficial functions to the body (66). In virtue of this and with the technological advance and consequent rise of knowledge and application of techniques based on genomic, metagenomic, and metabolomic study in recent years, microbial medicine has been developed and increasingly seen as an additive to conventional therapies (67). The admission of therapeutic methods such as the use of probiotics, prebiotics, and symbiotics aimed at GM modulation in the different types of disease associated with intestinal dysbiosis has represented a great advance for traditional medicine (60) and its effects have been widely reported in clinical and experimental studies (68, 69).

As described in the previous topic, intestinal dysbiosis is associated with the outcome and/or progression of various clinical situations. In this sense, a wide range of studies were developed to evaluate the cellular and molecular effects of these compounds in the treatment and prevention of the progression of various diseases. Thus, it was demonstrated that additive with prebiotic treatment was effective in reducing serum levels of pro-inflammatory cytokines IL-6 and TNF, indoxyl sulfate and urea nitrogen toxins, and the marker of oxidative stress malondialdehyde in the CKD patients subjected to dialysis (69). This study also reported that symbiotics were more efficient in reducing serum C -protein levels and endotoxin, while probiotics were better in relieving the gastrointestinal symptoms presented by these patients (69). In a double-blind and placebo-controlled clinical trial, it was shown that probiotic administration improved anthropometric parameters and triglyceride levels in obese patients (70). Corroborating these findings, in an experimental GM modulation study, it was shown that direct (via duodenal) and indirect (oral) intestinal microbial intervention is associated with improving the lipid and glycemic profile in rodents subjected to high fat diet, which are at least partly promoted by improving liver function (71).

Additionally, the beneficial effects of these or combined dietary supplements have been reported in numerous pathological contexts, including intestinal microbiota restoration of individuals with gastrointestinal disorders associated with infections or use of antibiotics (72), improvement of metabolic function in menopause women (73, 74) and the function of the thyroid in patients with disorders of this gland (74), protection against allergic response to ovalbumin in rats born by cesarean (68), decreased levels of muscle degradation markers, proinflammatory cytokines and sarcopenia in a mutual model of dexamethasone induced muscular atrophy (75) and improvement of synaptic plasticity, learning and memory in rats puppies submitted to a prenatal chronic stress protocol (76, 77).

Taking together, these data show that modulation of intestinal microbiota with food supplements is a viable and efficient alternative for promoting beneficial effects under the most diverse pathological conditions. This fact makes microbiota modulation an important target for future preventive or therapeutic interventions in more critical cases, for example, when organ transplantation is needed, since proven intestinal dysbiosis is associated, mainly, with allograft rejection in patients with solid organs such as liver, lung, heart and kidney (77). Moreover, although the beneficial role of these food supplements is well established in different types of disease, their influence as an adjuvant on substitutive therapy is still little explored, including kidney transplantation.

## Updates on microbiota and kidney disease/transplantation

CKD is a clinical condition characterized by a sustained reduction in kidney function and is considered a global public health problem (78). It affects about 10% of the world's population, which corresponds to approximately 850 million individuals (79) and is associated with high rates of progression to end-stage kidney disease (78). Although hypertension and diabetes are still the main causes of CKD development (3), intestinal dysbiosis has emerged as a potential driver not only of the development but also of the progression of CKD (67, 78). Nevertheless, in recent years the gut-microbiota-kidney communication has been extensively studied (20, 34, 78).

In this sense, it was shown that patients with stage 5 CKD, whether or not undergoing dialysis, presented an accumulation of serum levels of the uremic toxins indoxyl sulfate and p-cresyl sulfate associated with intestinal dysbiosis (80). More recently, it was demonstrated that the accumulation of these toxins was associated with loss of intestinal barrier function and consequently with greater progression of CKD (81). In fact, increased intestinal levels of indoxyl sulfate and p-cresyl sulfate are related to barrier disruption and intestinal leakage, which trigger systemic inflammatory cascades and consequently accelerate renal deterioration (17). These uremic toxins have a low molecular weight and are therefore easily removed by hemodialysis; however, their high binding affinity to serum albumin interferes with this clearance method, which can contribute to the development of uremic syndrome in these patients (82–84). More recently, it has been shown that both the total and free fractions of indoxyl sulfate and p-cresyl sulfate increase according to the stage of CKD, suggesting a new perspective for the use of these biomarkers as an indicator of CKD severity as well as for assessing renal function compared to current methods (83, 84). In addition to the accumulation of these toxins, chronic renal patients generally have a significant reduction in SCFA-producing bacteria such as *Faecalibacterium* and Lachnospira (80). It has been undisputedly demonstrated that patients with immunoglobulin A nephropathy (IgAN) and membranous nephropathy (MN) have a reduced abundance of microorganisms related to SCFA production, and this was directly associated with reduced levels of these SCFAs in these patients (78). Similarly, Song et al. (85) also demonstrated that elevated levels of gut bacteria, such as *Lachnospiraceae, Parasutterella, and Eubacterium*, and a consequent reduction in SFCAs levels are eventually related to a higher risk of developing diabetic kidney disease.

Like chronic renal patients, kidney transplant recipients also present intestinal dysbiosis (86) and in this case, the damage may be even greater, since it has been demonstrated that the change in GM is linked to acute graft rejection (87). In this sense, Fricke et al. (88) showed that specific modifications in the microbiota before kidney transplantation, *per se*, were enough to lead to adverse post-transplant events, including rejection and infectious complications. Subsequently, Lee et al. (86) demonstrated a significant reduction in bacteria from the Bacteroidetes phylum and an increase in *Pseudomonadota*, which is a phylum composed essentially of pathogenic bacteria, in kidney transplant patients, which in this case may increase the risk of complications in these post-transplant patients. Additionally, factors inherent to this type of procedure, such as immunosuppressive therapy and use of antibiotics after transplantation, have been linked to worsening intestinal dysbiosis, which in turn is associated with diarrhea, rejection, or dysfunction of the transplanted organ (89, 90).

Regarding immunosuppressive therapy, there is a bidirectional relationship between these medications and alterations in the GM (91). While these medications can alter the composition and diversity of GM, the immunosuppressants themselves can be affected by gains or losses in microbial taxa or specific functions of microorganisms, resulting in changes in their pharmacokinetics and/or pharmacodynamics (92, 93). The occurrence of these events, in turn, alters the metabolism of these drugs, favoring the presence of substances with a greater or lesser immunosuppressive effect compared to the original compound (94). Among the immunosuppressants used in clinical practice, the most well-known for affecting GM include corticosteroids, calcineurin inhibitors such as tacrolimus and cyclosporine, mycophenolate mofetil, and mTOR inhibitors (94). Mohamed et al. provides a current and comprehensive description of the pharmacokinetics and pharmacodynamics of all these immunosuppressants, focusing on the bidirectional relationship between these drugs and the gut microbiota (93).

## Microbiota modulation by synbiotics, probiotics, prebiotics, and high-fiber diets in renal allograft recipients

**Figure 1:**
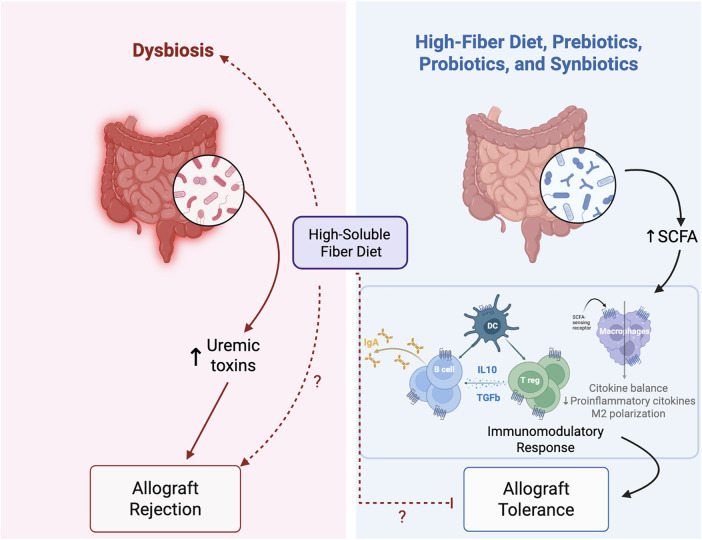
Gut microbiota effects on allograft outcomes: detrimental effects of dysbiosis versus protective effects of fiber-based and microbiota-targeted interventions. The left panel illustrates intestinal dysbiosis, characterized by an imbalance in gut microbial composition, often associated with increased intestinal permeability, which promotes increased production and systemic accumulation of gut-derived uremic toxins such as indoxyl sulfate and p-cresyl sulfate. These metabolites contribute to a proinflammatory milieu and are associated with progressive renal failure. This phenotype can persist even after kidney transplantation, damaging kidney epithelial cells and increasing the risk of allograft rejection. Conversely, certain dietary approaches have been explored by transplant recipients to improve transplantation outcomes. The right panel depicts the effects of a high-fiber diet, prebiotics, probiotics, and synbiotics in restoring eubiosis. Probiotics and synbiotics, in addition to other reported mechanisms (e.g., indole-3-aldehyde, bile acid, and nicotinamide metabolism), are frequently associated with the enrichment of SCFA-producing bacteria. Additionally, enhanced fermentation of dietary fibers present in high-fiber diets, prebiotics, and synbiotics increases the production of SCFAs such as acetate, butyrate, and propionate, which act on intestinal and systemic immune cells through known SCFA-sensing receptors (GPR41, GPR43, GPR109A, and olfactory receptor 78) to promote immunoregulatory pathways. SCFAs support dendritic cell (DC) tolerogenic functions, Treg expansion, IL-10 and TGF-*β* production, IgA responses by B cells, and macrophage polarization toward an anti-inflammatory M2 phenotype, while reducing proinflammatory cytokine expression. Collectively, these immunomodulatory effects favor cytokine balance and contribute to the establishment of allograft tolerance. However, it is important to note that recent studies have demonstrated that a high-soluble fiber diet may promote dysbiosis, colitis, and colorectal cancer. Given this, it is necessary to investigate whether the proportion of soluble and insoluble fibers may be critical for overall long-term outcomes. This concern is reflected by the question marks indicating unresolved effects of high-soluble fiber intake. The dashed arrows indicate proposed or incompletely defined mechanistic links between dysbiosis, microbial metabolites, and immune-mediated graft injury. Question marks denote pathways that remain incompletely understood and need investigation.

In recent years, concerns have arisen about the influence of diet on gut microbiota modulation and its effects on allograft tolerance. Diets and dietary components are shown to affect gut microbiome diversity. Consuming high-fiber diets, prebiotics, probiotics, and synbiotics has been used as a strategy to modulate microbiota after transplantation (Figure 1).

Synbiotics, which combine probiotics and prebiotics, have shown potential in restoring the gut microbiome and modulating immune responses. Randomized trials demonstrated that synbiotics could reduce serum uremic toxins as well as small intestine permeability, ameliorating gastrointestinal pain and constipation and favorably modifying the stool microbiome in CKD patients (95, 96). Similarly, a pilot study conducted by Guida et al. (97) found that synbiotics significantly reduced plasma levels of p-cresol in kidney transplant recipients. P-cresol is a uremic toxin that accumulates during chronic kidney disease and continues to be produced in excess after transplantation due to associated gut dysbiosis (95, 98, 99).

The findings suggest that the reduction in p-cresol levels observed after 15 and 30 days of synbiotic treatment may be due to the colonization of the gut by beneficial microorganisms present in the synbiotics (95, 97). These microorganisms lack the enzymes required for p-cresol production and instead produce lactic acid and SCFAs. This metabolic activity lowers intestinal pH, inhibiting the growth of bacteria like *Clostridium difficile*, which is known to produce p-cresol (100). However, another clinical trial suggests that long-term treatment with synbiotics may not improve serum uremic toxin levels and could actually decrease renal function in patients with CKD. This highlights the need for further studies in this area and raises questions about the duration of interventions (101) (Table 1).

**Table 1:** Summarized clinical trials with CKD or transplant recipient patients treated with synbiotics, prebiotics, or probiotics.

Study	Population	Intervention	Duration	Main outcome
Rossi et al. (95)	Predialysis adult participants with CKD (eGFR=10–30 mL/min per 1.73 m^2^)	Synbiotics: mixture of prebiotics (15 g/day across fructooligosaccharide, galactooligosaccharide, inulin) and probiotics[[4.5 × 10^10^ CFU/day (nine strains across Lactobacillus, Bifidobacteria, Streptococcus)]	Two periods of 6 weeks with 4-week washout	Reduction in serum p-cresyl sulfate without significantly change free indoxyl-sulfate and modification of stool microbiome with enrichment of with enrichment of *Bifidobacterium* and depletion of *Ruminococcaceae.*
Cosola et al. (96)	Stage IIIb-IV CKD Patients	Synbiotics [NATUREN G®: mixture of probiotics (*Lactobacillus Casei* LC4P1 2.4 × 10^9^, *Bifidobacterium Animalis* BLC1 2.4 × 10^9^), prebiotics (fructoligosaccharides 2.5 g and inulin 2.5 g) and natural antioxidants (a mix of quercetin 0.064 g, resveratrol 0.023 g and proanthocyanidins 0.013 g)]	2 months	Reduction in serum free indoxyl-sulfate levels and in small intestinal permeability with amelioration of abdominal pain and constipation syndromes in CKD patients.
Guida et al. (97)	Kidney transplant recipients	Synbiotics [Probinul Neutro, CadiGroup, Rome, Italy: lyophilized bacteria (5 × 10^9^ *Lactobacillus plantarum*, 2 × 10^9^ L*actobacillus casei subsp. rhamnosus*, 2 × 10^9^ *Lactobacillus gasseri*, 1 × 10^9^ *Bifidobacterium infantis*, 1 × 10^9^ *Bifidobacterium longum*, 1 × 10^9^ *Lactobacillus acidophilus*, 1 × 10^9^ *Lactobacillus salivarius*, 1 × 10^9^ *Lactobacillus sporogenes*, and 5 × 10^9^ *Streptococcus thermophilus*), prebiotic inulin (2.2 g; VB Beneo Synergy 1, Beneo Iberica, Barcelona, Spain) and 1.3 g of tapioca-resistant starch]	30 days	Reduction in plasma p-Cresol concentration after 15 and 30 days of symbiotic treatment.
McFarlane et al. (101)	Adult participants with CKD (eGFR=15–60 mL/min per 1.73 m^2^)	Synbiotics: mixture of prebiotics [20 g/day of high-resistant starch fiber supplement (Hi-Maize 260, 50% resistant starch; Ingredion)] and probiotics [4.5 × 10^11^ colony-forming units (CFU)/day of nine different strains from three different genera (Bifidobacteria, Lactobacillus, and Streptococcus; Swiss Mendes)]	12 months	Increased serum creatinine levels, reduced eGFR, and modification of stool microbiome with enrichness of *Bifidobacterium* and *Blautia spp.*
Zhang et al. (107)	Liver transplant recipients	Probiotics (*Bifidobacterium lactis*, *Lactobacillus acidophilus*, *L. plantarum*, *L. casei*, *L. rhamnosus*, and *L. brevis*)	At least 7 days	Reduction in postoperative bacterial infections (22%) and the duration of antibiotic use.
Kujawa-Szewieczek et al. (108)	Hospitalized patients receiving antibiotics after organ transplantation or immunosuppressive therapy for any other reason in the nephrology and transplantation ward	Probiotic [Sanprobi IBS®: one capsule contains at least 10 × 10^9^ colony forming units (CFU) of *Lactobacillus plantarum 299v*]	Two twelve-months period observation	Reduction in the incidence rate of *Clostridium difficile* infections from 12.1 to 1.1 per 1,000 patients.
Chan et al. (111)	Kidney transplant recipients	Probiotic: *Lactobacillus plantarum* MFM 30-3 (5 × 10^9^ CFU), *Lactobacillus paracasei* MFM 18 (5 × 10^9^ CFU) and Orafti P95 (Oligosaccharides 10 mg, Microcrystal-line *α*-Cellulose 300 mg, Magnesium Stearate 300 mg, Silicon Dioxide 300 mg)	3 months (2 pills daily during the first month, then 1 pill daily during the second and third months)	Improved renal function by lowering creatinine levels and increasing eGFR.
Chan et al (117); Chan et al. (105)	Kidney transplant recipients	Prebiotic (green banana–resistant starch)	7 months	Reduction in gastrointestinal symptoms (less frequent abdominal pain and reflux); and improvement in gut microbial diversity and richness
Yoshifuji et al. (125)	Allogeneic hematopoietic stem cell transplantation recipients	Prebiotics [8 g cornstarch containing 70% resistant starch (Amylofiber SH; J-Oil Mills Inc, Tokyo, Japan) for lunch and dinner; and a mixture of 3 g glutamine, 5 g polydextrose, and 1.45 g lactosucrose (Otsuka Pharmaceutical Factory Inc, Tokushima, Japan) at the breakfast]	From the start of pretransplantation conditioning regimen until day 28 after the transplantation	Reduced incidence and severity of acute graft-versus-host disease, shorter period of oral mucositis and diarrhea, and the maintenance of microbiota diversity.

Additionally, *Clostridium difficile* infection is a prevalent concern for patients following solid organ transplants and during immunosuppressive therapy, often linked to episodes of antibiotic-related diarrhea (10, 100, 102, 103). Some studies suggest that synbiotic and probiotic supplementations may effectively reduce bacterial infections following transplantation (104–106). A study conducted by Zhang et al. (107) demonstrated that the use of probiotics, including *Bifidobacterium lactis*, *Lactobacillus acidophilus*, *L. plantarum*, *L. casei*, *L. rhamnosus*, and *L. brevis*, resulted in a 22% reduction in bacterial infections among liver transplant recipients. Furthermore, incorporating *Lactobacillus plantarum 299v* during antibiotic treatment has been shown to decrease the incidence of *Clostridium difficile* infections, highlighting its potential prophylactic benefits (108, 109).

Probiotics, such as *L. casei Zhang*, *Lactobacillus johnsonii*, and *Bacteroides ovatus*, have also been associated with reduced renal inflammation and fibrosis, as well as enhanced intestinal barrier function, demonstrating the potential use of probiotics for preventing CKD, and maybe for kidney transplant recipients as has been suggested for peritoneal dialysis by Stepanova (110). Additionally, Chan et al. (111) showed in a pilot study that supplementation with *Lactobacillus plantarum* and *Lactobacillus paracasei* improved renal function in kidney transplant recipients with stabilized graft function. These beneficial effects are mediated by several mechanisms (110), which reinforce the importance of investigating the gut-kidne*y* axis. The probiotic *L. casei Zhang* slows the progression of kidney disease in mice and patients with CKD by increasing SCFAs and through nicotinamide metabolism, which decreases renal inflammation by modulating local macrophages and tubular epithelial cells (112). The negative correlation between the abundance of *Lactobacillus johnsonii* and CKD progression demonstrated by Miao et al. (113) suggests it could be a potential target for reversing CKD by suppressing the aryl hydrocarbon receptor (AHR) signal via indole-3-aldehyde (IAId) serum levels. Additionally, the oral administration of *Bacteroides ovatus* protects against renal fibrosis and restores intestinal barrier function in CKD models (114). These benefits are mediated by the growth of *Clostridium scindens* in the gut, which produces hyodeoxycholic acid and stimulates the intestinal production of glucagon-like peptide 1 (GLP-1). This, in turn, activates GLP-1 receptors in the kidneys, contributing to the protective effects of *Bacteroides ovatus* (114). Despite that and the increasing prescription of probiotics for pediatric kidney transplant recipients (109, 115), a consensus on their safety and efficacy for the pediatric population has yet to be established (116). Typically, these prescriptions address concerns related to antibiotic-associated diarrhea, *Clostridium difficile* infections, and the promotion of overall gastrointestinal health (116).

Prebiotics, on the other hand, are fibers that the human body cannot digest, but the microorganisms in our gut can break down and utilize. A randomized clinical trial demonstrated a reduction in gastrointestinal symptoms, as shown by a lower frequency of reflux and abdominal pain, following prebiotic supplementation with green banana-resistant starch in kidney transplant recipients (105, 117). Moreover, an improvement in gastrointestinal microbiome diversity was observed, indicated by an increase in microbial richness and Shannon diversity (118).

Furthermore, fiber intake has been proven to lower the risk of post-transplant metabolic syndrome (118, 119). Moreover, CVD associated with metabolic syndrome are among the main causes of diabetes and kidney allograft loss after transplantation (119–122). Additionally, high-fiber diets have been linked to an increase in beneficial *Bacteroidetes* and *Prevotella*, while reducing opportunistic bacteria like *Bacillota* (123). Also, it prevents dysbiosis associated with transplantation (124, 125). Conversely, the Western diet, which is high in fat and processed sugar but low in fiber, promotes the growth of opportunistic bacteria (123).

The modulation of the microbiome through a high-fiber diet is frequently associated with an increase in SCFAs like acetate, butyrate, and propionate. These SCFAs are essential for the health benefits provided by high-fiber diets (108, 123, 126). Similarly, it has been observed that *Lactobacillus plantarum 299v* ingestion can also increase these SCFAs in the feces of healthy volunteers (108). Additionally, in animal studies involving rats, butyrate has been shown to protect kidneys from injury caused by ischemia-reperfusion (127, 128) —a process that occurs in transplanted kidneys due to ischemic conditions during retrieval, preservation, engraftment, and subsequent reperfusion (34). Reducing renal ischemia-reperfusion injury is crucial for the success of kidney transplants, as it can lead to either acute or chronic rejection of the kidney allograft (129).

Recent research has also highlighted the significant role of gut microbiome metabolites in modulating the immune system across various contexts (45, 130). In the field of cancer immunology, metabolites such as butyrate, propionate, and acetate have been demonstrated to enhance the antitumor activity of natural killer cells (131). Moreover, formate has been shown to promote the proliferation and functionality of CD8 ^+^ T cells in antitumor responses (132). Furthermore, sodium acetate supplementation, similarly to a high-fiber diet (guar gum together with cellulose), has been linked to improved tolerance of kidney allografts, protecting against both acute and chronic rejection (124, 133). This effect is mediated by the induction of regulatory T cell development through GPR signaling pathways (124, 134). Additionally, intestinal metabolites from the microbiota are connected to the generation and maintenance of regulatory B cells, which help reduce skin allograft rejection (129).

On the other hand, Yang et al. (135) demonstrated that a high soluble fiber diet promotes colorectal tumorigenesis in mice, even when mixed with insoluble fiber. The excess of high-soluble fibers, such as inulin and guar gum, modulates the gut microbiota, inducing dysbiosis with the enrichment of *Bacteroides uniformis* and depletion of *Bifidobacterium pseudolongum*. This dysbiosis is accompanied by metabolic changes, including elevated serum bile acids, increased fecal butyrate, and lower levels of inosine, effects not observed with a diet rich in insoluble fiber, such as cellulose (135). Similarly, another recent study found that processed partially hydrolyzed guar gum increases susceptibility to colitis and colon tumorigenesis in mice (136). While studies in humans have yet to validate this hypothesis, it is important to consider how the amount of soluble fiber in the diet may influence the long-term benefits of high-fiber and prebiotic diets.

## Conclusion

In summary, these findings suggest that gut dysbiosis is linked to allograft rejection. Although more research is necessary about the use of high-fiber diets, prebiotics, probiotics, and synbiotics in transplanted patients, especially concerning efficacy and safety in the pediatric population, it seems that modulating the gut microbiome through the consumption of short-chain fatty acids-producing diets could have therapeutic implications by enhancing immunomodulatory responses and improving transplantation outcomes. On the other hand, recent studies have related the use of high-soluble fiber to increased susceptibility to colitis and colon tumorigenesis in animal models. However, no observational studies have been conducted in humans yet. In the same direction, the SINERGY II clinical trial suggests that long-term synbiotics treatment could facilitate renal failure in CKD patients, which highlights the need for larger clinical trials and a better understanding of the correlation between the time of intervention and the type of pre-, pro-, and synbiotics utilized.

Considering that transplant recipients are already at heightened risk for malignancies due to chronic immunosuppression, it is important to take into account the potential risks of these kinds of interventions. Additionally, the DIGEST study, whose protocol has been published by Singer et al. (137), may provide the first insight into the effects of inulin, a high-soluble fiber, in kidney transplant recipients. However, they will follow up with the patients only for 12 weeks after transplantation, demonstrating the need for studies with longer follow-ups. Therefore, future studies and larger clinical trials are also necessary to determine whether fiber subtypes can interfere with the effects observed in kidney allograft tolerance and post-transplant malignancies in the long term.
